# Biofilm Producing *Salmonella* Typhi: Chronic Colonization and Development of Gallbladder Cancer

**DOI:** 10.3390/ijms18091887

**Published:** 2017-08-31

**Authors:** Enea Gino Di Domenico, Ilaria Cavallo, Martina Pontone, Luigi Toma, Fabrizio Ensoli

**Affiliations:** 1 Clinical Pathology and Microbiology, San Gallicano Institute, Istituto di Ricovero e Cura a Carattere Scientifico (IRCCS), 00144 Rome, Italy; ilaria.cavallo90@gmail.com (I.C.); martina.pontone@ifo.gov.it (M.P.); fabrizio.ensoli@ifo.gov.it (F.E.); 2 Infectious Disease Consultant, Regina Elena National Cancer Institute, Istituto di Ricovero e Cura a Carattere Scientifico (IRCCS), 00144 Rome, Italy; luigi.toma@ifo.gov.it

**Keywords:** biofilm, *Salmonella* Typhi, gallbladder cancer, skin manifestations, toxin, DNA Damage Response, infection, inflammation, gallstone

## Abstract

*Salmonella enterica* subspecies *enterica* serovar Typhi is the aetiological agent of typhoid or enteric fever. In a subset of individuals, *S*. Typhi colonizes the gallbladder causing an asymptomatic chronic infection. Nonetheless, these asymptomatic carriers provide a reservoir for further spreading of the disease. Epidemiological studies performed in regions where *S.* Typhi is endemic, revealed that the majority of chronically infected carriers also harbour gallstones, which in turn, have been indicated as a primary predisposing factor for the onset of gallbladder cancer (GC). It is now well recognised, that *S.* Typhi produces a typhoid toxin with a carcinogenic potential, that induces DNA damage and cell cycle alterations in intoxicated cells. In addition, biofilm production by *S.* Typhi may represent a key factor for the promotion of a persistent infection in the gallbladder, thus sustaining a chronic local inflammatory response and exposing the epithelium to repeated damage caused by carcinogenic toxins. This review aims to highlight the putative connection between the chronic colonization by highly pathogenic strains of *S*. Typhi capable of combining biofilm and toxin production and the onset of GC. Considering the high risk of GC associated with the asymptomatic carrier status, the rapid identification and profiling of biofilm production by *S*. Typhi strains would be key for effective therapeutic management and cancer prevention.

## 1. Epidemiology and Risk Factors

*Salmonella enterica* subspecies *enterica* serovar Typhi is a rod shaped, flagellated, aerobic, Gram-negative bacterium. It is a human-specific pathogen that causes typhoid or enteric fever [1]. The most frequent cause of infection by *S*. Typhi is the consumption of contaminated food or water. After ingestion, *S*. Typhi establishes a systemic infection by invading the mucosal surface of the intestine, spreading into the liver, spleen, pancreas and bone marrow [2]. The multi-systemic complications associated with *S*. Typhi infection can lead to a fatal outcome if diagnosed late or not effectively treated. 

The major symptom of the disease is a non-specific fever [2], which increases during the first week of the illness, generally reaching a steady phase in the second week with temperatures around 39–40 °C, with few diurnal variations.

Other common symptoms include chills, headache, malaise, anorexia, a dry cough, sore throat, and myalgia, thus resembling more common seasonal viral infections. The presence of skin manifestations is not unusual, characterized by the presence of a non-itching erythematous maculopapular rash [3,4,5], generally located on the chest, abdomen and occasionally on the back, arms, legs and genitals [1,6]. 

*S.* Typhi causes 22 million cases of typhoid fever, 5.4 million cases of paratyphoid fever and 216,000 deaths worldwide annually [7,8]. In regions where *S.* Typhi is endemic approximately 1–4% of infected individuals become chronic asymptomatic carriers representing a threat to local public health [9,10]. Chronic infection with *S.* Typhi, which is generally localized in the gallbladder, is classically associated with long-term excretion of bacteria [11]. Although carriers have frequent biliary tract disease, this condition is not thought to be necessary for development of the carrier status [12,13,14]. Epidemiological studies performed in *S.* Typhi endemic regions, such as Chile, Bolivia, Ecuador, as well as some areas of India, Pakistan, Japan and Korea, have shown that approximately 90% of chronically infected carriers are also gallstones carriers, and this association is, in turn, indicated as a major predisposing factor for the development of gallbladder cancer (GC) [15,16,17,18,19]. 

GC is the sixth most common gastrointestinal tract cancer and represents one of the most widespread biliary tract malignancies [20]. The worldwide annual incidence of GC is approximately 2/100,000 individuals, with marked ethnic and geographical variations [21]. The highest incidence rates were found among South Americans, in American–Indians, in the north of India, in Pakistan and Korea. In several European and North American countries, GC is rare and mortality is declining, although relatively high incidence and mortality rates are still reported in some central European countries [20]. The malignancy has been associated with genetics and life style, but *S.* Typhi infection and gallstone disease represent the most important risk factors [22]. Gallstone size increases the risk of GC. When stones exceed 3 cm the risk is tenfold higher compared with smaller stones [23,24].

The survival rate, after five years is reported to be around 30% for lesions confined to the gallbladder mucosa but this percentage drops to 10% after one year for more advanced stages of the disease [25]. Although the interplay of multiple factors is likely to contribute to the development of the malignancy, the association with a chronic *S.* Typhi infection has been observed and repeatedly confirmed since 1971 [26].

Strong epidemiological evidence of the association between *S.* Typhi infection and GC came from retrospective studies conducted in the United States and in Europe, proving that chronic *S*. Typhi carriers were exposed to a significantly higher risk of death from GC in comparison with otherwise healthy individuals [27]. Typhoid and paratyphoid carriers, enrolled in the study during the typhoid outbreak of 1994 in Scotland, showed a greater probability of GC, and to a lesser extent, pancreas, colo-rectum and lung cancer as well as other less frequent neoplasms [15]. Subsequent studies confirmed these observations, linking typhoid carriers with an increased incidence of cancer of the hepatobiliary system, although lacking any serological evidence [28]. Conversely, serological studies conducted in northern India, showed that the rates of *S.* Typhi isolation from bile, gallbladder tissue and gallstones from patients with GC were significantly higher as compared to those of patients suffering from benign gallbladder diseases [16,29,30]. The frequencies of individuals having clinically significant anti-Vi serum antibody titres were 38.5% for GC patients, 13.9% for patients with benign gallbladder diseases, and 9.2% for healthy individuals. More recently, a specific nested PCR technique, developed to overcome the limitations of serology test and culture isolation, revealed the presence of the *S.* Typhi flagellin gene in 67.3% of hepatobiliary samples collected from GC patients while in patients with benign gallbladder diseases and healthy population this percentage was significantly reduced [17,31]. 

The hypothesis of a causative link between *S.* Typhi and GC has been reinforced by several studies performed in Chile that present incidence rates of GC of 12.8/100,000 cases in women and 6.3/100,000 in men, which are among the highest in the world [27]. This study, which involved culture assessment and quantitative PCR, showed a positive correlation between *S.* Typhi and GC, suggesting that the early detection of *S*. Typhi is critical in order to develop prevention strategies for gallbladder carcinogenesis [27].

Histologically, over 90% of gallbladder cancers are adenocarcinoma and more than 80% of cases are also associated with the presence of gallstones [25,32]. Indeed, strong epidemiological evidence correlates the presence of GC with the chronic *S*. Typhi carrier status in association with gallstones [19,33].

Although a positive correlation between *S*. Typhi and GC is well documented, the mechanism(s) for its chronic persistence as well as the putative carcinogenic mechanisms promoted by *S*. Typhi are still debated. The production of biofilm may exert a key role in supporting the colonization and chronic persistence of *S*. Typhi. This notion is supported by several reports documenting that the bile, a lipid-rich, detergent-like digestive secretion with antimicrobial properties contained in the gallbladder, induces the production of an exopolysaccharide matrix O-antigen that facilitates *S.* Typhi biofilm formation on human gallstones [34,35]. Thus, biofilm embedded gallstones may represent a most favourable environment for bacterial persistence in the gallbladder and may lead to reseeding of the intestine and faecal shedding, with the ensuing transmission to a new host, while chronically exposing the gallbladder epithelium to bacterial factors with potential carcinogenic properties.

## 2. Potential Carcinogenic Activity of *S.* Typhi

Different products released by *S.* Typhi have the potential to promote carcinogenesis including bacterial glucuronidase and nitroso compounds [25]. In addition, *S.* Typhi also produces a toxic molecule, belonging to the group of the cytolethal distending toxins (CDT), which induces DNA damage and triggers an irreversible cell cycle arrest and apoptosis [36]. The presence of CDT has been identified in several Gram-negative bacterial species including: *E. coli*, *Campylobacter* spp., *Shigella dysenteriae*, *Actinobacillus actinomycetemcomitans*, *Haemophilus ducreyi* and numerous enterohepatic *Helicobacter* spp. [37]. CDTs are normally encoded by three different genes, *cdtA*, *cdtB*, and *cdtC*, organized as an operon encoding three polypeptides (CdtA, CdtB, and CdtC), which are assembled as a heterotrimeric toxin [37,38,39]. This molecule has an active unit called CdtB which possesses structural and functional homology with the mammalian DNAse-I [40,41]. Several studies reported that CdtB causes double stranded breaks in host cell chromosomal DNA, activating the DNA damage response (DDR) [36,42,43]. 

CDT toxic activity was first identified from clinical isolates of *Escherichia coli* [44]. In vitro experiments demonstrated that this toxin triggers cell cycle arrest and ultimately cell death in some cultured mammalian cells [45]. In the tripartite toxin complex, the CdtB is the active subunit that functions as a DNAse I, while CdtA and CdtC mediate the binding of the holotoxin to the plasma membrane of the target cells. Once CdtB penetrates into the target cells it induces limited double strands breaks (DSB), leading to activation of the ataxia-teleangiectasia mutated (ATM)-dependent DNA damage responses and the formation of DNA repair complexes [42,46]. *S.* Typhi represents an exception to the conventional CdtA–CdtB–CdtC tripartite structure characteristic of the CDT family [40,47]. It possesses an open reading frame (ORF) encoding a protein with a high amino acid sequence similarity to CdtB but does not present an ORF with amino acid sequence similarity to CdtA or CdtC [40]. Hence, CdtB is not associated with CdtA and CdtC, but its activity relies on the expression of two genes, resembling the B components of pertussis toxin, named pertussis-like toxin A (*pltA*) and pertussis-like toxin B (*pltB*). The structure of typhoid toxin revealed an A2B5 organization with the CdtB and PltA covalently linked in the A subunit and associated with the PltB homopentamer forming the B subunit. The C-terminal region of PltA forms an alpha-helix inserted into the channel formed by the PltB pentamer maintained by hydrophobic interactions [48]. 

The primary target for typhoid toxin binding are glycoproteins, mediated by the PltB pentamer [48,49]. Typhoid toxin binds to different glycans, exhibiting a specific preference for those with terminal sialic acids. Human sialoglycans differ from those present in other mammals, representing a central factor in the S. Typhi’s host specificity [49].

**Figure 1:**
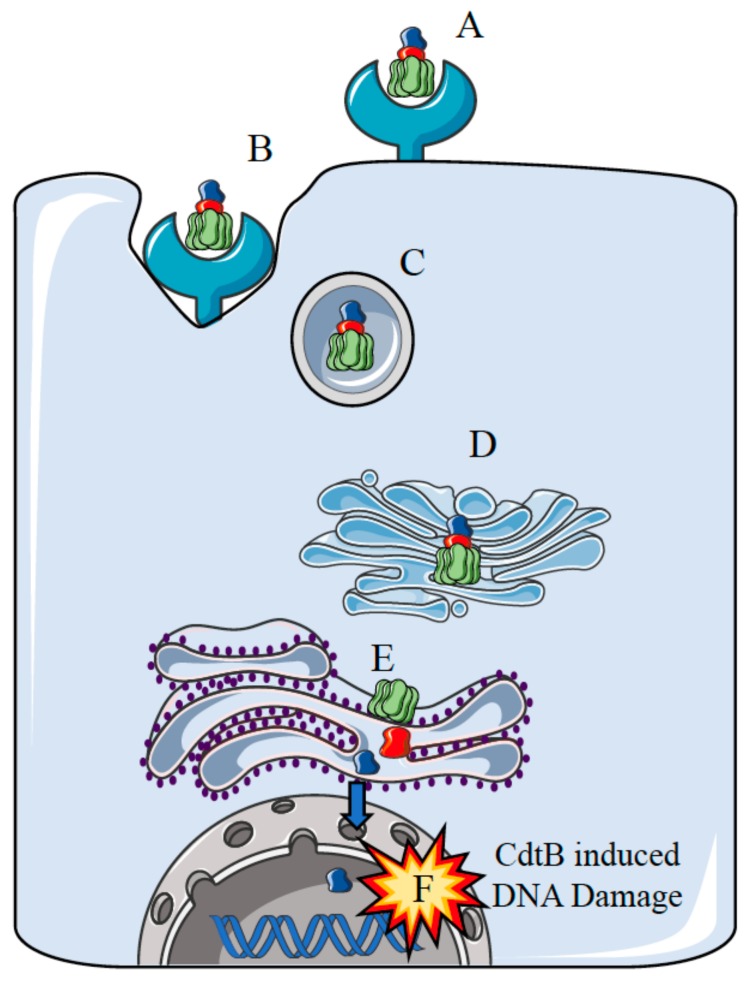
Proposed model for the intracellular trafficking of the typhoid toxin. (**A**) The PltB subunits bind to the host cell surface receptor; (**B**) Endocytosis of the typhoid toxin; (**C**) Endosome-mediated delivery of the typhoid toxin to the Golgi complex; (**D**) Passage of the typhoid toxin through the Golgi complex; (**E**) Entry of the typhoid toxin into the endoplasmic reticulum (ER) and release of the CdtB component from the holotoxin (**F**) Entry of the CdtB subunit into the nucleus and induction of DNA damage [46,47,48].

**Figure 2:**
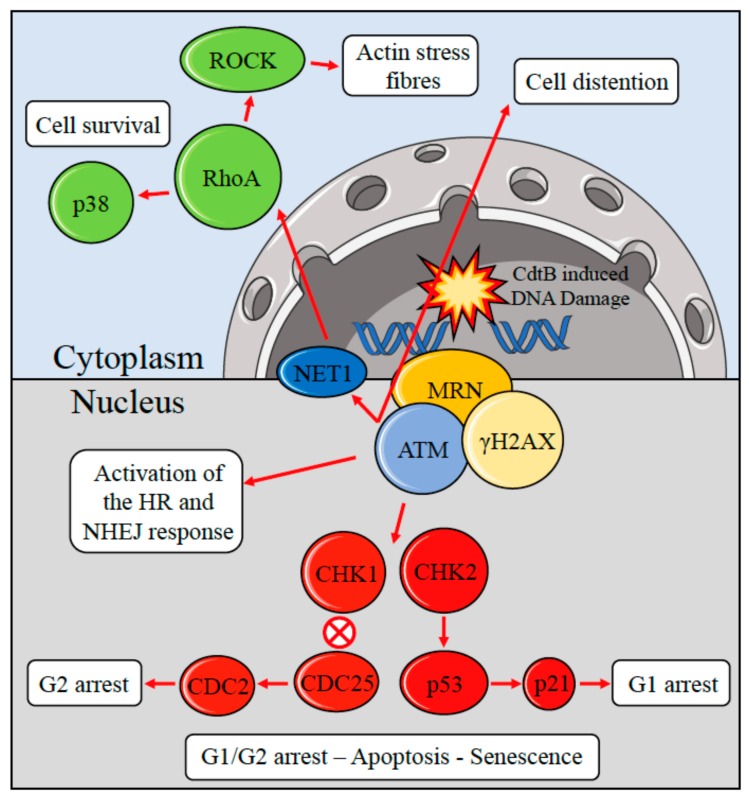
CdtB induced double strand breaks and activation of the ataxia-teleangiectasia mutated (ATM) signalling network. ATM is recruited and fully activated to the site of double-strand break (DSB) by the Mre11-Rad50-Nbs1 (MRN) complex. Adjacent to the site of damage, ATM phosphorylates the histone H2AX (γH2AX) which in turn promotes chromatin remodeling and ATM retention at the site of damage. ATM phosphorylates and activates the downstream effector checkpoint kinases 1 (CHK1) and 2 (CHK2). CHK1 activation induces the accumulation and stabilization of p53 which transcriptionally activates the target gene p21. CHK2 phosphorylates and inhibits the cell division cycle 25 (CDC25), leading to the downstream hyperphosphorylation and inactivation of the cyclin dependent cell division cycle 2 (CDC2) kinase. The checkpoints modulation, mediated by ATM, induces cell cycle arrest either in the G1 or G2 phases of the cell cycle to allow the DNA damage repair, alternatively, when the damage cannot be repaired, this response drives the cell to apoptosis or cellular senescence. The ATM signaling in the cytosol activates the Ras homolog gene family, member A (RhoA). This process results in the activation of the RhoA/Rho-associated, coiled-coil containing protein kinase 1 (ROCK) axis which modulates the organization of the actin cytoskeleton, and in the activation of the p38-mediated cell survival pathway.

The *S*. Typhi CdtB–PltA–PltB tripartite complex, after being delivered to the target cell, reaches the nucleus of the target cell inducing DNA damage [36,40,50]. The mechanism of action of the tripartite complex reflects the specific mode of infection of *S.* Typhi. Expression of *cdtB*, *pltA*, and *pltB* take place only after bacterial internalization into host cells [36,40]. When *S.* Typhi reaches an intracellular compartment, it releases the CdtB–PltA–PltB complex into the vacuole [36,40]. The complex is then transported to the plasma membrane and secreted to the extracellular medium from where it can target neighbouring non-infected cells inducing DNA damage and G2 cell cycle arrest [36,40,41,48]. Entry of the typhoid toxin into target cell occurs via endocytosis (Figure 1). The PltB and PltA subunits are probably required to transport CdtB from the site of production within the cells to the external cellular compartment [51]. This finding originated from the observation that during bacterial infection of tissue culture cells, the CdtB toxin can exert its effects on both infected and non-infected cells, inducing cell cycle arrest and cellular distension [40]. Comparative studies, based on CDTs produced by other bacterial species, suggest that the typhoid toxin may follow an endocytic trafficking similar to that described for other AB-type toxins. This would result in typhoid toxin internalization by endocytosis, transport to the Golgi complex and then to the endoplasmic reticulum (ER) [41,52,53,54,55,56]. Subsequently, the activated CdtB subunit translocates to the host cell nucleus inducing DNA damage [53]. The activity of CdtB originates from toxin-mediated DNA damage, which activates the cellular DNA damage response. Indeed, CdtB possesses catalytic residues with eukaryotic DNase I, activities, suggesting that this protein might function as an intracellular-acting DNase [42,57,58,59,60]. In vitro studies also indicated that CdtB acts as a nuclease cleaving DNA substrate, inducing nuclear fragmentation and chromatin disruption either in mammalian cells or in *Saccharomyces cerevisiae*, and promoting DNA fragmentation in cells exposed to the purified soluble toxin [42,57,61,62]. The DNA damaging activity of the CdtB toxin activates a cascade of events (Figure 2) which include the classical DNA damage response, with the recruitment of the DNA damage sensor complex Mre11-Rad50-Nbs1 (MRN), which initiates DNA end resection to produce a 3′ tail with the accumulation and full activation of the ATM kinase at the site of the damage [63]. As a consequence, activation of ATM promotes phosphorylation of histone H2AX, a member of the histone 2A family and activation of several DNA damage checkpoints including the tumour suppressor p53 and its downstream effector p21, which induces cell cycle arrest in G1 [64]. Additionally, ATM activates the checkpoint kinases 2 (CHK2), that causes inactivation of the cell division cycle 25 (CDC25) phosphatase. CHK2-dependent inactivation of the CDC25 leads to an accumulation of the hyperphosphorylated form of the cyclin-dependent checkpoint kinases 1 (CDK1) that, in turn, blocks cell proliferation in the G2/M phase [46,65,66,67,68]. In some cases, CdtB-exposed normal or tumour cells showed 53BP1⁄cH2AX-positive foci, senescence associated b-galactosidase activity, and expansion of promyelocytic nuclear compartments that represent hallmarks of transition into cellular senescence [69]. Thus, the carcinogenic potential of the genotoxins released by *S.* Typhi can also be indirectly linked to the activation of survival signalling pathways that induces cells with DNA damage to acquire genomic instability and mutations by activating the DNA damage checkpoint responses. In fact, the survival of cells accumulating DNA damage and with genomic instability is considered to be a major cause of cancer [70]. 

The survival of cells exposed to CDT relies on the activation of the small GTPase Ras homolog gene family, member A (RhoA), which promotes the formation of actin stress fibres [62]. This process is not directly caused by the toxin, but is the consequence of the ATM-induced cellular responses to genotoxic stresses. Activation of RhoA, mediated by the ATM kinase, prevents cell death via activation of p38 and its downstream target mitogen-activated protein kinase-activated activated protein kinase 2 (MK2) prolonging cell survival [71]. Indeed, factors that disrupt cell cycle progression may induce the acquisition of mutations. This might be achieved by a mechanism analogous to genotoxic agents such as Reactive Oxigen Species (ROS) and Reactive Nitrogen Species (RNS), that increase the rate of mutations within the genome [72,73]. Therefore, CdtB-induced activation of survival responses in cells harbouring DNA damage could contribute to the accumulation of genetic instability within the developing inflammatory microenvironment, providing the ideal background for promoting the transformation of pre-neoplastic cells to malignant cells in the host. To what extent the activation of the DDR by the typhoid toxin provides an evolutionary advantage to *S*. Typhi is unclear. *S*. Typhi is strictly a human pathogen and evaluation of the direct effect of the typhoid toxin in vivo is a challenging task. In a recent publication, immunocompetent mice, with an unperturbed microbiota, were orally infected with isogenic *S*. *enterica*, serovar Typhimurium strains, producing either a functional or an inactive typhoid toxin. In this model, the typhoid toxin was detected ten days post-infection in the liver of infected mice. Surprisingly, it was found that typhoid toxin favored the survival of the host, promoting a significant reduction of severe enteritis in the early phases of infection. Subsequent analysis confirmed that the functional typhoid toxin suppressed the intestinal inflammatory response and increased the frequency of asymptomatic carriers [74,75]. Induction of DNA damage and activation of ATM by oxidative burst, suppress cytokine production in purified human neutrophils activated by bacterial elements (LPS flagellin and zymogen) [76]. From an evolutionary prospective, the activation of the DDR by the typhoid toxin may suppress the inflammatory response in support of a chronic infection. In this scenario, carcinogenesis could represent collateral damage of the prolonged release of the typhoid toxin. In addition to the typhoid toxin, recent studies showed the ability of *S. enterica* to induce cellular transformation through the activation of host pathways capable of promoting bacterial uptake and its intracellular survival [77]. This mechanism is mediated by the activation of the host Mitogen-activated protein kinase (MAPK) and by the AKT pathway, which regulate cellular proliferation, growth, and apoptosis [77,78]. Invasion of epithelial cells by *S. enterica* is promoted by type III secretion system (T3SS-1) encoded by *S. enterica* pathogenicity island 1 (SPI1) and the T3SS-1 secreted bacterial effectors SopB, SopE, SopE2, SptP and SipA required for invasion and entry into cells [79]. Bacterial effectors activate MAPK and AKT pathways which in turn induce the transformation of cells harboring inactivated p53 and overexpressing the MYC oncogene [77], as observed in tissue samples of GC tumors isolated from Indian patients infected by *S.* Typhi [77,80].

## 3. Biofilm-Mediated *S*. Typhi Persistence in the Gallbladder

The establishment of *S*. Typhi chronic carrier status is associated in 80–90% of cases with the presence of cholesterol gallstones [81]. On the other hand, the simultaneous presence of *S.* Typhi and gallstones is associated with increased risk for the development of GC [16,82,83]. The use of high-dose, prolonged antibiotic treatment is not always capable of eradicating *S*. Typhi colonization of the gallbladder in chronically infected patients, although partial efficacy has been observed in patients without gallstones [12,82]. Although cholecystectomy has been proved to increase the rate of resolution, additional sites of infection have been observed in the liver, biliary tree and mesenteric lymph nodes [18,25]. The chronic persistence, the difficulty in the therapeutic eradication of *S*. Typhi colonization together with its ability to evade the host immune response in carriers, strongly suggests the presence of a typical biofilm-related microbial disease [18]. Indeed, previous reports have shown the presence of biofilm-coated gallstones in asymptomatic human carriers, in mouse models and on the gallbladder epithelium of mouse carriers, providing direct evidence that biofilm production by *S*. Typhi may play a pivotal role for the establishment of a chronic colonization in the gallbladder [18,33,84,85]. Gallbladder tissues from patients undergoing cholecystectomy showed the presence of *S*. Typhi colonization and the microscopic observation of gallstones revealed a consistent biofilm formation on their surface [86,87]. Enterobacteriaceae, different from *S*. Typhi, have frequently been recovered from gallbladder tissues and gallstones [88,89]. In the latter, however, gallstones are not massively covered by bacterial biofilm as in the case of *S*. Typhi, suggesting that *S*. Typhi may have adopted a specific strategy, which relies on biofilm production on the gallstone’s surface, to chronically persist in the gallbladder and support the continuous microbial shedding and reattachment process, followed by bacterial diffusion via urine and faeces, as observed in chronic carriers [33,84,90].

This notion is reinforced by in vivo studies performed in a mouse model fed with a lithogenic diet (1% cholesterol and 0.5% cholic acid), to promote the development of cholesterol gallstones. The results showed an enhanced colonization of the gallbladder as compared to infected mice lacking gallstones [33]. Additionally, the infected mice with gallstones had an increased level of faecal shedding of *S.* Typhimurium, as compared with infected mice lacking gallstones. The subsequent microscopic analysis of the gallstones showed the presence of a dense bacterial biofilm covering the surface [33].

Colonization of the gallbladder exposes *S.* Typhi to bile, a complex digestive secretion containing bile acids, cholesterol, phospholipids and bilirubin, that possesses strong antimicrobial properties [91]. *S.* Typhi tolerates this apparently hostile environment indicating that bile resistance is a central pathogenetic process in both acute and chronic gallbladder infection [34]. Furthermore, bile can also induce pleiotropic responses in *Salmonella* spp, as well as in other enteric pathogens, such as *Campylobacter jejuni* [92], *Escherichia coli* [93], *Listeria monocytogenes* [94] and *Vibrio cholerae* [95], regulating the expression of numerous genes [18]. Interestingly, it has been previously demonstrated that *S*. Typhi forms biofilm on human gallstones and cholesterol-coated Eppendorf tubes in a process mediated by the presence of bile [34,90]. Indeed, bile exerts a profound effect on direct or indirect gene regulation in *S*. Typhi [34,96,97] by downregulating the expression of genes involved in host cell invasion and motility and outer membrane proteins [34,84,98,99]. Indeed, the notion that biofilm-producing *S.* Typhi can successfully adapt to the gallbladder environment is further reinforced by clinical evidence showing that *S.* Typhi isolates from gallbladder carriers exhibit a common genetic profile, distinct from strains infecting other organ sites [100], with specific phenotypic traits including an increased biofilm forming ability [19,87]. On the other hand, it has been shown that *Salmonella* colonization can be compromised by infiltrating cells that do not produce biofilm together with biofilm producing bacteria. In fact, the infiltration with biofilm non-producer cells gave rise to a delayed and milder disease development in the murine model, which was accompanied by a higher susceptibility to antibacterial agents [101]. Thus, biofilm formation appears to be a key adaptive strategy adopted by *S.* Typhi in order to allow microbial persistence, sustaining an increased resistance against antibiotics and the host immune response while ensuring microbial spreading in the host and the community. Chronic persistence represents a key element for prolonging the mutagenic effect of bacterial toxins on target cells, which may lead to cumulative damages and transformation.

## 4. Conclusions

**Figure 3:**
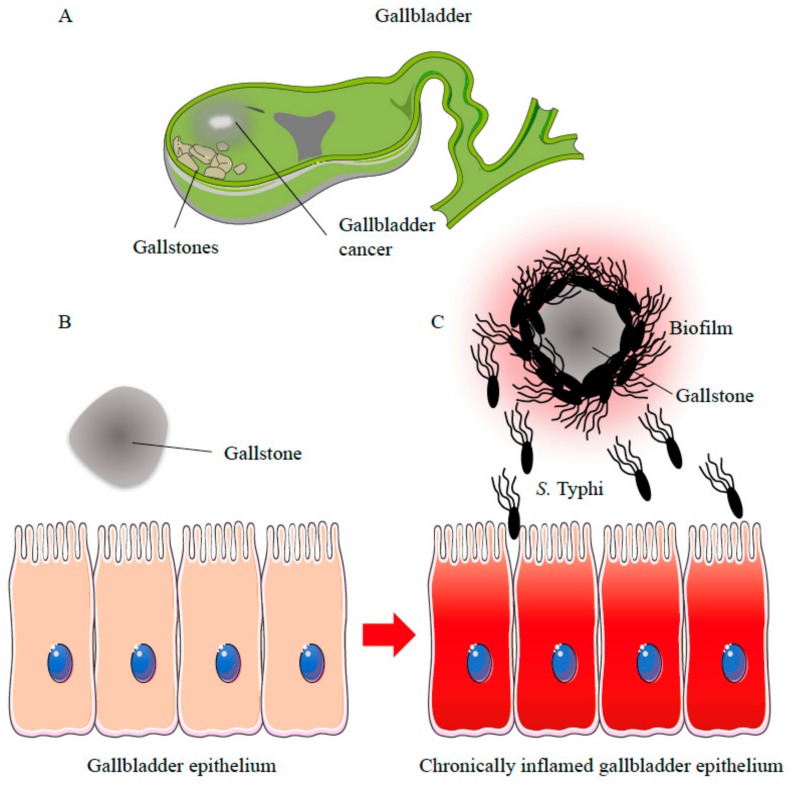
Possible role of biofilm-producing *S*. Typhi in gallbladder cancer development. (**A**) Chronic infection with *S*. Typhi strains and the presence of gallstones strongly correlates with gallbladder cancer (GC) development; The presence of gallstones (**B**) may provide the ideal substrate for *S*. Typhi strains with an increased biofilm forming ability; (**C**) Once the biofilm is established bacterial cells can detach from the gallstones releasing carcinogenic molecules that induce genomic instability and chronic inflammation which represent key prerequisites for the onset of GC.

Gallbladder colonization and chronic persistence of *S*. Typhi appears to be favoured by biofilm formation, which is most effective on the surface of the gallstone. Several studies have described the strong association between the development of a chronic carrier status and the presence of gallstones. On the other hand, biofilm formation on gallstones has also been consistently observed and bile and cholesterol have been found to enhance bacterial adhesion [18,84]. Nevertheless, chronic *S*. Typhi infection is still considered as the primary predisposing factor for the development of GC in individuals with specific predisposing factors, independently from the presence of gallstones. Although, in vivo and in vitro evidence has demonstrated that *S*. Typhi can form biofilm on gallstones and that the presence of biofilm on gallstones is a hallmark of typhoid carriage, the putative link between the prevalence of GC and biofilm coated gallstones has been not adequately explored so far. In addition to gallstones, other sites, such as the gallbladder epithelium, should be considered as alternative niches for *S*. Typhi chronic infection [18]. Additional studies will be required to investigate the possible association between biofilm-producing *S*. Typhi, chronic carriers with gallstones and the onset of GC. Characterization of the molecular mechanisms involved in biofilm formation on gallstones and the activity exerted by *S*. Typhi in promoting gallbladder inflammation and damage also remain to be further investigated. The chronic production of genotoxins and free radicals with immunomodulatory properties may further induce DNA damage and mutations in gallbladder tissue. The presence of the CDT complex, which induces DNA damage as well as the activation of MAPK and AKT pathways, indicates that *S.* Typhi can promote cell transformation and GC particularly in genetically predisposed individuals. Thus, *S*. Typhi may be responsible for stable transformative effects in the host cells through a multi-step process involving colonization, chronic persistence and toxin production (Figure 3).

It has been proposed that bacterial degradation of bile salts and other bacterial byproducts could cause GC [83]. This mechanism resembles that recently proposed for intestinal bacteria with the potential to induce colon cancer (CC) by the release of polyamine metabolites. Interestingly, in this process biofilm plays a central role by upregulating polyamine metabolites that affect the growth of cancer cells, and creating favorable conditions for oncogenic transformation in colonic epithelial cells. [102,103]

Additional studies should aim at evaluating strategies for the prevention of biofilm formation on the gallstone surface. New clinical laboratory tests to assess biofilm formation as well as to determine the antibiotic resistance profiles of biofilm-forming bacteria should be introduced. The identification of high-risk infections, such as those sustained by strong biofilm producing *S*. Typhi, may support the most effective therapeutic interventions for microbial eradication [104]. Antibiotic treatment selected on the basis of the conventional antimicrobial susceptibility testing are frequently ineffective in the eradication of bacteria associated in biofilm [105]. Once the biofilm is established, the individual cells exhibit an increased tolerance to antimicrobial agents and the antibiotic treatment alone is often inadequate. Within a biofilm matrix, microbial cells show 10–1000 times higher minimal inhibitory concentration (MIC) than the same bacterial cells examined in planktonic growth conditions [106,107]. The effective antibiotic MIC in vivo for biofilm eradication may be impossible to achieve by the administration of antibiotics at doses that appear effective in planktonic growth, due to the toxicity and the side effects of the drugs.

Our preliminary results, aimed at evaluating the level of biofilm production by the clinical Biofilm Ring Test (cBRT), revealed that biofilm is a major virulence determinant in chronic skin ulcers and it is critical in the failure of the antibiotic treatment in patients with medical devices-related infections [108,109,110]. The timely recognition of a high biofilm producer, before the development of a mature biofilm matrix, may help direct a more appropriate targeting of the therapeutic intervention (type, doses, duration) and decision-making (e.g. catheter removal). In the case of *S*. Typhi the rapid identification of strong biofilm-producing strains may identify high risk patients, thus supporting clinicians in the prevention of microbial-associated cancers.
